# 18β-Glycyrrhetinic Acid Delivered Orally Induces Isolated Lymphoid Follicle Maturation at the Intestinal Mucosa and Attenuates Rotavirus Shedding

**DOI:** 10.1371/journal.pone.0049491

**Published:** 2012-11-13

**Authors:** Jay M. Hendricks, Carol Hoffman, David W. Pascual, Michele E. Hardy

**Affiliations:** Department of Immunology and Infectious Diseases, Montana State University, Bozeman, Montana, United States of America; Massachusetts General Hospital, United States of America

## Abstract

Glycyrrhizin, an abundant bioactive component of the medicinal licorice root is rapidly metabolized by gut commensal bacteria into 18β-glycyrrhetinic acid (GRA). Either or both of these compounds have been shown to have antiviral, anti-hepatotoxic, anti-ulcerative, anti-tumor, anti-allergenic and anti-inflammatory activity *in vitro* or *in vivo*. In this study, the ability of GRA to modulate immune responses at the small intestinal mucosa when delivered orally was investigated. Analysis of cytokine transcription in duodenal and ileal tissue in response to GRA treatment revealed a pattern of chemokine and chemokine receptor gene expression predictive of B cell recruitment to the gut. Consistent with this finding, GRA induced increases in CD19^+^ B cells in the lamina propria and B220^+^ B cell aggregates framed by CD11c^+^ dendritic cells in structures resembling isolated lymphoid follicles (ILF). Using a mouse model of rotavirus infection, GRA reduced the duration of viral antigen shedding, and endpoint serum antibody titers were higher in GRA-treated animals. Together the data suggest GRA delivered orally augments lymphocyte recruitment to the intestinal mucosa and induces maturation of B cell-rich ILF independently of ectopic antigenic stimulus. These results provide further support a role for dietary ligands in modulation of dynamic intestinal lymphoid tissue.

## Introduction

Pharmacologically active constituents in extracts of the medicinal licorice root include glycyrrhizin (GA) and its aglycone metabolite 18β-glycyrrhetinic acid (GRA). Both compounds have been extensively studied for their effects on cellular physiology and as immune system modulators in cultured cell lines, in small animal models and in humans, with either or both demonstrating anti-tumorgenic, anti-allergenic, anti-hepatotoxic, antiviral, anti-ulcerative, or anti-inflammatory properties (reviewed in [1]). Multiple mechanisms of activity have been proposed including inductive or inhibitory effects on apoptosis, cytokine expression, intracellular signaling pathways, transcription factor activation, cellular membrane fluidity and modulation of oxidative stress [1]–[6]. How or if these mechanisms function *in vivo* to account for the ability of these compounds to attenuate pathology in infectious and inflammatory diseases is not well understood.

GA has been shown to be beneficial *in vivo* in several systems. In the clinical setting, intravenous administration of a commercial formulation containing GA (Stronger Neo-Minophagen®) has been used in Japan for >20 years to treat patients with chronic viral hepatitis, with evidence of clinical improvement and reduction in progression to hepatocellular carcinoma [7]–[10]. Murine models of infectious and inflammatory diseases provide further evidence for immune modulating or antimicrobial properties of GA. GA reduces lethality associated with influenza virus infection [11], and attenuates carrageenan-induced lung injury [4], LPS-induced acute respiratory stress syndrome [12], and OVA-induced allergic asthma [13]. In the gut, GA and a formulation called Si-Ni-San containing GA, ameliorate inflammation-mediated pathology in a mouse model of colitis [14], and are associated with decreased expression of proinflammatory cytokines IFN-γ, IL-12, TNF-α, and IL-17, and increased expression of anti-inflammatory cytokines IL-10 and TGF-β. GA-induced anti-inflammatory cytokine expression also was demonstrated in a gut ischemia-reperfusion model [15].

In contrast to GA, less *in vivo* data are available for GRA. Despite less direct evidence for *in vivo* activity, GA is rapidly metabolized into GRA [16], and it is likely that some of the immune modulating effects of GA are attributable to its primary metabolite. Studies have shown intraperitoneal administration of GRA to mice in a model of visceral leshmaniasis results in reduced parasite burden [17], and repeated subcutaneous administration of GRA abrogates lung pathology associated with *Staphylococcal* pneumonia [18]. In addition, we recently have shown that GRA reduces lesion size and virulence gene expression in a mouse model of MRSA skin infection [19]. Taken together, these studies provide evidence that GA and GRA modulate immune responses to a variety of infectious agents, and regulate cell stress responses in chronic inflammatory environments, suggesting potential of these purified compounds to be used as therapeutics or immune adjuvants. There are little data however, that address whether these compounds have similar activity when taken orally, and whether purified compounds or crude extracts commonly used as dietary supplements affect host defense responses through this route of administration.

In this study, potential mechanisms of immune system modulating activity of orally administered GRA were investigated. Analysis of cytokine gene expression in small intestinal tissue following administration of GRA revealed a specific pattern of chemokine and chemokine receptor gene expression that was predictive of B cell recruitment to the gut mucosa. Increases in CD19^+^ B cells in the small intestinal lamina propria were observed in GRA-treated mice, and histological analyses identified B220^+^ B cell clusters with morphology and cell content consistent with structures of isolated lymphoid follicles (ILFs). The ability of GRA to induce lymphoid tissue maturation independently of ectopic antigenic stimulus suggests GRA affects immune cell responses in the gut and activates signaling pathways favorable to modulation of mucosal B cell populations. Using the adult mouse model of rotavirus infection, we further show that GRA shortened the duration of viral antigen shedding, suggesting the changes in gene expression and lymphocyte recruitment to the intestine induced by GRA likely is functionally relevant in enteric virus infection.

## Materials and Methods

### Ethics Statement

All animal experiments were performed according to the NIH Guidelines for Care and Use of Animals, with approval from the Montana State University Institutional Animal Care and Use Committee (Protocol number 2011-44).

### Compounds and Virus

Glycyrrhizin (GA) and 18β-glycyrrhetinic acid (GRA) were purchased from Sigma-Aldrich. Stock solutions were prepared to a concentration of 100 mg/mL in DMSO (vehicle) and aliquots were stored at −80°C. Stock solutions were diluted to working concentrations in calcium-magnesium free phosphate-buffered saline (PBS), and tested for endotoxin with the Limulus Amoebocyte Lysate Assay (Associates of Cape Cod, Inc). The final concentration of endotoxin in the working stock was <0.025 EU/dose.

Murine rotavirus strain EW was prepared and maintained in intestinal homogenates harvested from neonatal mice as previously described [20].

### Animal Dosing and Infections

Four to six week old male C57BL/6 mice were obtained from Jackson Laboratories. Fifty mg/kg of GRA or vehicle only was administered by oral gavage according to the timetable dictated by the experiment. For infection studies, mice were administered 10^5^ shedding dose 50 (SD_50_) of EW in a volume of 100 µL by oral gavage, or 100 µL of intestinal homogenate prepared from uninfected neonatal mice. Fecal samples were collected daily. Animals were euthanized at the conclusion of the experiments to harvest intestinal tissue for RNA isolation, flow cytometry, and histology.

### Cytokine Arrays

Following oral administration of GRA, one cm sections of either duodenum or ileum were dissected and stored in RNAlater (Qiagen) at 4°C for a minimum of 18 hrs. All sections were devoid of Peyer’s Patches. RNA was extracted with the RNeasy system (Qiagen) and quantified with a Nanodrop 1000 (Fisher Scientific). Cytokine transcripts were measured with the SABiosciences Mouse Inflammatory Cytokine Array (PAMM-011A) or Custom Mouse RT^2^ Profiler™. Custom arrays included Cxcr5, Ccl19, Ccl21b, Cxcl13, Lta, Ltb, Ccr6, Ccr7, Ccr9, Ifng, and Il10. One µg of RNA was reverse transcribed with RT^2^ First Strand kit (SABiosciences) following the manufacturer’s instructions. PCR reactions were performed on an Realplex 4 s (Eppendorf ). Reaction conditions consisted of 95°C for 10 minutes, followed by 40 cycles of 95°C for 15 seconds, and 60°C for one minute. Data from a minimum of three mice per group were combined and are expressed as fold-change over vehicle-treated animals. Fold-changes >2 were scored as significant.

### Harvesting and Analysis of Intestinal Cell Populations

At the indicated times post-dosing and/or post-infection, cells from the Peyer’s Patches (PPs), mesenteric lymph nodes (MLNs), and small intestinal lamina propria (LP) were isolated as previously described [21]. Antibodies used for staining and analysis by flow cytometry included: anti-CD4 A488, anti-CD8 PE, anti-CD19 PE-cy7, anti-CD69 eF605, anti-CD127 PE-cy5, anti-CD185 PE, and anti-CD8a AF700, all from eBiosciences. Anti-CD138 PE and anti-CD11c APC were from BD Biosciences. Flow cytometry was performed on a BD LSR flow cytometer using FacsDIVA software and data were analyzed with FlowJo software.

### ELISAs for Fecal Rotavirus Antigen Shedding and Anti-rotavirus Serum Antibody

ELISA for fecal rotavirus antigen detection was performed as previously described [22]. Fecal samples were diluted 10-fold w/v in TNC (50 mM Tris, 150 mM NaCl, 5 mM CaCl_2_) containing 0.05% Tween-20 and protease inhibitors (25 µM leupeptin, 1.5 µM aprotinin, 1 mM benzamidine, 30 µM pepstatin A). Flat-bottom 96-well plates were coated with a monoclonal antibody to rotavirus structural protein VP6 (A6M) [23] diluted in carbonate/bicarbonate buffer overnight at room temperature. 50 µL fecal suspension was added to the wells and plates were incubated for one hour at 37°C. Anti-rotavirus SA11 antibody was added to the wells and incubated for one hour at 37°C, followed by HRP-conjugated goat anti-rabbit antibody.

To detect serum antibody to rotavirus [22], 96 well plates were coated with anti-SA11 antibody overnight. SA114F stock virus was treated with 25 mM EDTA for 20 minutes, then added to the wells and incubated for one hour at 37°C. Serial dilutions of serum samples were added to the wells and incubated for an additional hour at 37°C. Reactions for both the fecal antigen ELISA and the serum antibody ELISA were developed with TMB Microwell Peroxidase (KPL) for 10 minutes, then quenched with 1 M H_3_PO_4_. Absorbance at a wavelength of 450 nm was measured on a VersaMax Microplate Reader (Molecular Devices).

### Histology

Small intestine was dissected and separated into duodenum, jejunem and ileum. The lumen of each section was rinsed with PBS, and then infused with OCT. The sections were coiled into a cryomold with the proximal end at the center, covered with OCT and snap frozen in liquid nitrogen. Five µm thick sections were mounted on Superfrost slides (Fisher), and fixed with 75% acetone/25% ethanol for five minutes, air dried and then stained with antibodies to B220 (A488), CD11c (PE), and CD3e (PE), all from eBiosciences. Images were captured on a Nikon Eclipse i80 fluorescent microscope. Mean fluorescence intensity (MFI) was measured with NIH Image/ImageJ software (http://rsb.info.nih.gov/nih-image/about.html).

## Results

### GRA Induces Transcription of a Specific Pattern of Genes Encoding Chemokine Receptors and Corresponding Ligands in the Small Intestine

The ability of orally delivered GRA to modulate immune system activity at the gut mucosa initially was analyzed by measuring cytokine gene expression in small intestinal tissue. Mice were administered GRA or vehicle alone by oral gavage. Ten hours post-treatment, total RNA was extracted from sections of the gut taken ∼one cm from the gastroduodenal junction, or the ileum, and changes in cytokine gene transcription were measured by RT-qPCR using a Mouse Inflammatory Cytokine array. In the initial experiment, ten genes were up-regulated in GRA-treated mice >2-fold over vehicle-treated controls. These genes, listed in Table 1, plus one additional gene of interest, CCL21b, were chosen to design custom arrays. The same pattern of up-regulated chemokine and chemokine receptor transcripts was observed in multiple repetitions of the experiment, and was similar regardless of whether RNA was extracted from duodenal or ileal tissue. GRA-induced transcripts included chemokine receptor CXCR5 and its ligand CXCL13, receptor CCR7 and its ligands CCL19 and CCL21b, and receptors CCR6 and CCR9. Increased transcription of genes encoding the ligands for CCR6 and CCR9 (CCL20 and CCL25, respectively) did not meet the established cut-off of >2-fold change, although CCL20 was moderately up-regulated in the original full array (1.6 fold, data not shown). Lymphotoxin A (Lta) and lymphotoxin B (Ltb) also were up-regulated in GRA-treated animals. IFN-γ and IL-10 were moderately increased in the first experiment, but induction was not consistent between multiple experiments. Some variability between mice in terms of the presence or absence of a response was observed. However, the pattern was reproducible with respect to both the transcripts that were induced and the relative magnitude of expression in all mice that responded, and these genes never were up-regulated in vehicle-treated controls. Expression of genes encoding these chemokine receptors and their corresponding ligands is consistent with signals known to be required for lymphocyte recruitment to the intestinal mucosa, and with formation and maturation of B cell-rich isolated lymphoid follicles (ILF, ([24], [25], see below).

**Table 1:** GRA-induced changes in cytokine expression in intestinal tissue.

	Duodenum	Ileum	+EW
Cxcr5	30.0	21.0	23.4
Ccl19	3.4	15.7	13.8
Ccr6	9.4	12.5	9.8
Ccr7	5.3	10.4	6.4
Ccr9	2.6	2.2	1.3
Cxcl13	4.2	4.6	7.1
IFNγ	2.6	1.4	ND
Il10	2.6	1.6	1.3
Lta	6.8	8.5	4.3
Ltb	4.2	6.2	4.1
Ccl21b	ND	3.6	2.3

Representative data are shown for RNA isolated from duodenal or ileal tissue. Data shown for duodenal tissue are from the initial full array. Data from ileal sections from uninfected and EW infected mice were obtained with the custom array. Data are presented as fold-increase over mock-treated controls. ND – not done.

To test whether enteric rotavirus infection affected induction of these genes by GRA, mice were infected for 18 hours with murine rotavirus strain EW prior to administration of GRA. The same pattern of gene expression was observed (Table 1), indicating virus replication does not modulate the signal-inducing activity of GRA early post-infection. These results suggest GRA likely has a direct effect on specific cellular targets in the small intestinal mucosa that results in coordinated chemokine and receptor gene expression.

### Immune Cell Populations Induced in MLNs and PPs by GRA

**Figure 1:**
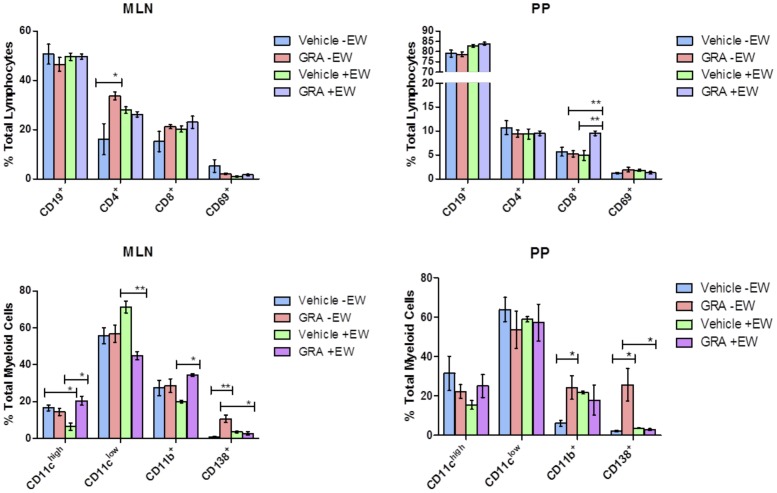
Immune cell populations modulated by GRA in uninfected and rotavirus -infected mice. C57Bl/6 mice (n = 5 per group) were administered GRA or vehicle alone orally one day pre-infection with 10^5^ SD_50_ of murine rotavirus strain EW, and then one day post-infection. Cells isolated from the MLNs and PPs were analyzed for changes in B cells (CD19), T cells (CD4 and CD8), their activation (CD69); and dendritic cells (CD11c^high^ and CD11c^low^), macrophages (CD11b), and plasma cells (CD138). *p<0.05, **p<0.01. Error bars are SEM.

The observed pattern of chemokine and receptor gene expression led us to examine the effects of GRA on immune cell populations at mucosal inductive sites. Mice were administered GRA or vehicle and infected with EW or mock-infected. Animals were sacrificed nine days post-infection and immune cell populations in the PPs and MLNs were analyzed by flow cytometry (Figure 1). The percentage of CD4^+^ T cells increased in GRA-treated, uninfected mice compared to vehicle-treated controls in the MLNs, but not in the PPs. In the PPs, CD8^+^ T cells were significantly increased in GRA-treated, infected mice relative to vehicle-treated, infected mice. CD8^+^ T cells also appeared to increase in the MLNs in GRA-treated, uninfected mice compared to vehicle-treated animals, but this increase did not score as significant. These data suggest GRA may have an effect on T cell accumulation in these inductive tissues, particularly CD8^+^ T cells in PP of infected mice. Analysis of myeloid cell populations in GRA- or vehicle-treated, infected animals showed significant differences in dendritic cell (DC) subsets CD11c^high^ and CD11c^low^, as well as macrophage (CD11b^+^) cell populations in the MLNs. The only significant difference observed in the PPs was CD11b^+^ cells in GRA treated, uninfected mice.

**Figure 2:**
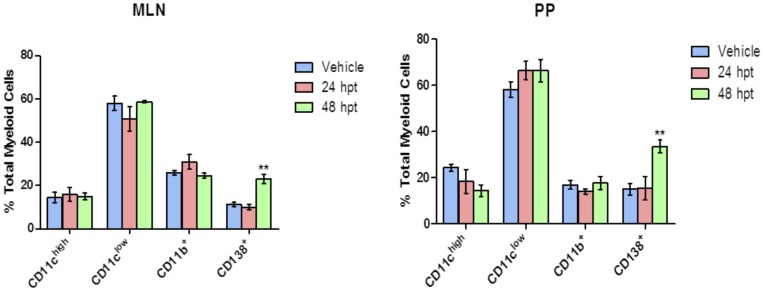
CD138^+^ cells are increased in MLNs and PPs 48 hours post-treatment (hpt) with GRA. Mice (n = 3 mice per group) were administered GRA or vehicle by oral gavage, and cell populations in the MLNs and PPs were analyzed with antibodies to CD11c, CD11b, and CD138. **p<0.01. Error bars are SEM.

A striking difference in the CD138^+^ population was observed between mice given GRA and mice administered vehicle. CD138 (syndecan-1) is expressed on pre-B and immature B cells in the bone marrow, absent on circulating B cells, and re-expressed on plasma cells [26]. GRA-treated mice had a significantly higher percentage of CD138^+^ cells than vehicle-treated mice both in the MLNs and the PPs (Figure 1). This difference was not observed in GRA-treated infected mice, likely overshadowed by influx of lymphocytes into these tissues in response to virus infection. To investigate this further and determine the kinetics of the initial response, mice (uninfected) were gavaged with GRA or vehicle, and MLNs and PPs were harvested 24 and 48 hours post-treatment (Figure 2). CD138^+^ cells were increased in both tissues by 48 hours in animals given GRA, but not in animals given vehicle, suggesting GRA affects B cell differentiation in these mucosal inductive sites.

### GRA Induces CD19^+^ B Cell Recruitment to the LP

**Figure 3:**
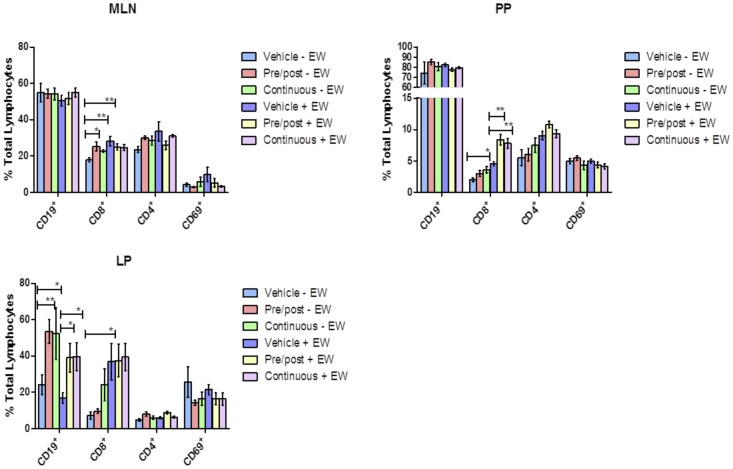
GRA induces CD19^+^ cell accumulation in the lamina propria in uninfected and rotavirus infected mice. Mice (n = 5 mice per group) were administered GRA or vehicle by oral gavage, and then mock-infected or infected with EW. Two dosing schedules were used: 1) GRA or vehicle alone was administered one day pre-infection and then one post-infection (pre/post), or 2) every two days through the course of infection. Nine days post-infection, cell populations isolated from the MLNs, PPs and LP were analyzed by flow cytometry for changes in B (CD19^+^) and T (CD4^+^ and CD8^+^) cells. *p<0.05, **p<0.01. Error bars are SEM.

To test how the timing of GRA dosing affected B and T cell populations in mucosal inductive sites as well as in the LP effector site, mice were treated either one day pre-infection and one day post-infection (or mock-infection) as before, or every other day for the course of the experiment. In the MLNs, significant increases in the CD8^+^ T cell population in GRA-treated, uninfected mice relative to vehicle-treated controls were observed (Figure 3). There were no differences in CD4^+^ or CD8^+^ T cell populations between the different dosing schedules. In PPs, there were no significant differences in CD4^+^ T cells between GRA-treated and vehicle-treated uninfected or infected animals, except the overall percentages in infected mice were somewhat higher. In contrast, CD8^+^ T cells in the PPs markedly increased in GRA-treated, infected animals compared to animals given vehicle, as before. A similar trend was observed in uninfected animals, although this difference was not as great. These data further support the idea that GRA impacts CD8^+^ T cells in mucosal inductive tissues. Importantly, changes in CD8^+^ T cells were not enhanced by repeated GRA doses, suggesting the initial signaling events induced by GRA are of key importance and result in a sustained cellular immune response.

LP lymphocytes also were analyzed to determine whether orally delivered GRA could influence immune cell populations at a mucosal effector site. A profound increase in CD19^+^ B cells was observed in the LP of GRA-treated mice (Figure 3). Importantly, this increase was observed in both uninfected and infected animals, and there were no differences between the dosing schedules. These data, interpreted in the context of the gene expression data, suggest GRA induces B cell recruitment to the small intestinal mucosa, and does so in the absence of ectopic antigenic stimulus.

### GRA Induces Formation of B220^+^ B Cell Clusters Resembling ILF

ILFs consist of a single B cell follicle surrounded by DC with few scattered T cells [27], [28]. In an experiment prompted by gene expression data and demonstrated increases in CD19+ cells in the LP, mice were administered GRA or vehicle, and then mock-infected or infected with rotavirus to determine if ILFs were induced by GRA. GRA was given one day pre-infection and then again one day post-infection, as before. Intestinal sections were prepared one day following the second dose of GRA, or nine days post-infection and stained for detection of B cells (B220), DCs (CD11c), or T cells (CD3).

**Figure 4:**
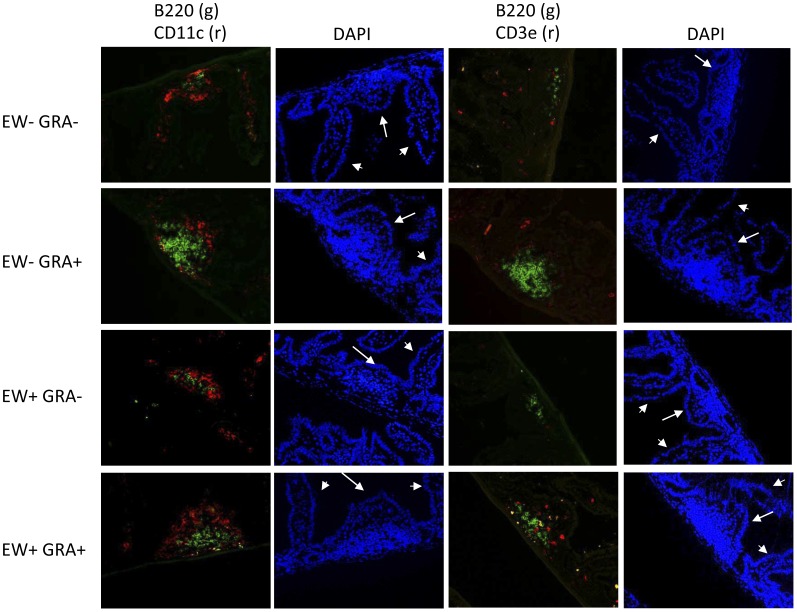
GRA induces formation of B220^+^ aggregates in uninfected and rotavirus-infected mice. Mice (n = 3 mice per group) were administered GRA or vehicle by oral gavage, and infected or mock infected with EW. In these mice, GRA or vehicle was administered one day pre-infection and then again one day post-infection. Ileal sections were prepared one day after the second GRA dose and then were stained for the detection of B cells (B220), DCs (CD11c), and T cells (CD3). Arrows indicate ILF-containing villi; arrowheads indicate adjacent ILF-absent villi. Magnification = 20×.

In ileal sections harvested one day after the second dose of GRA, obvious B220^+^ cell clusters surrounded by CD11c^+^ cells, and a few CD3^+^ T cells were observed (Figure 4). DAPI staining further indicated that villi containing these B220^+^ cell clusters were shorter and broader than surrounding villi, and typical LP structure was displaced. This cell composition and the morphology of villi where they are located are consistent with mature ILF [27], [29]. These structures were not present in tissues from vehicle-treated mice. Instead, small areas of CD11c^+^ cells with few B220^+^ cells were routinely visible. Estimates of changes in B220^+^ cell density between GRA-treated and vehicle-treated animals made by measuring mean fluorescent intensity indicated a ΔMFI >7 fold between the two groups. The smaller structures in vehicle-treated mice may be indicative of immature ILF [28], [29], and suggest GRA induces ILF maturation and ectopic antigenic stimulus is not required.

**Figure 5:**
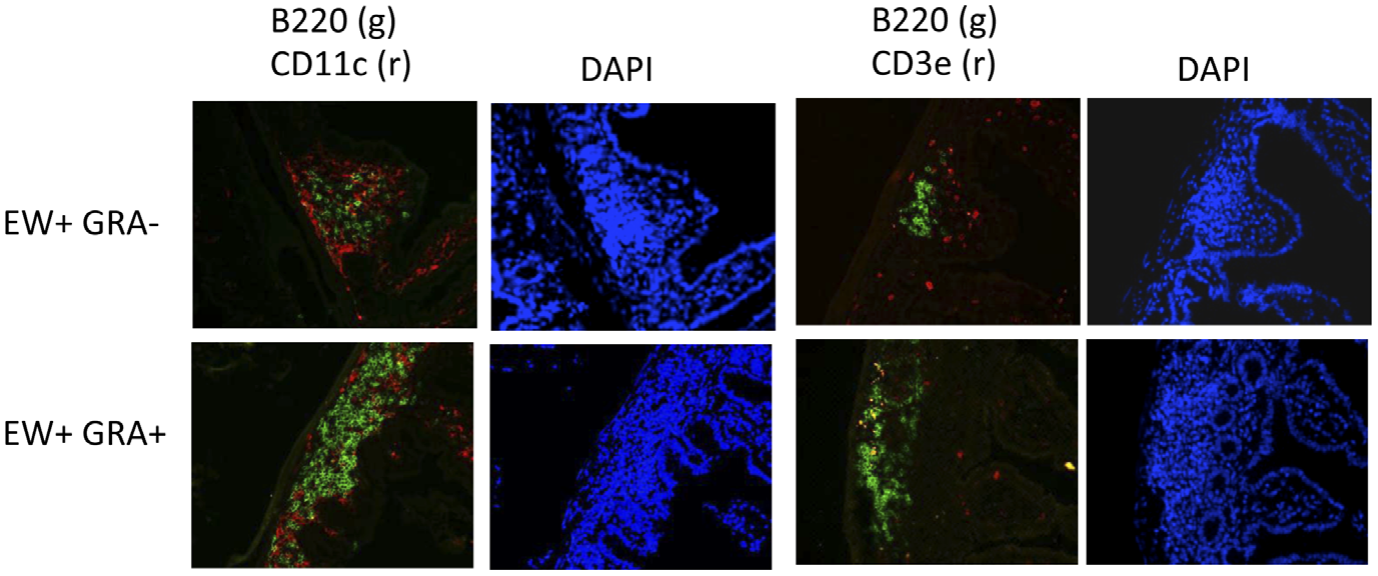
GRA enhances formation of B220^+^ aggregates in rotavirus-infected mice at 9dpi. Mice (n = 3 mice per group) were administered GRA or vehicle by oral gavage, and infected or mock infected with EW. In these mice, GRA or vehicle was administered one day pre-infection and then again one day post-infection. Ileal sections were prepared nine days post-infection, and then were stained for the detection of B cells (B220), DCs (CD11c), and T cells (CD3). Arrows indicate ILF-containing villi; arrowheads indicate adjacent ILF-absent villi. Magnification = 20×.

In sections harvested from infected mice at the early time point, B220^+^ cells were increased in GRA-treated mice relative to vehicle-treated mice (ΔMFI >3 fold, Figure 4). B220^+^ cells also appeared increased in GRA-treated infected mice at nine days, at which time the infection was resolved, although the difference was not as great (ΔMFI >1.5 fold, Figure 5). These data suggest rotavirus infection induces ILF which has not been observed before, and that GRA might augment the B cell response in the gut mucosa.

### Oral Administration of GRA Reduces the Duration of Rotavirus Antigen Shedding

**Figure 6:**
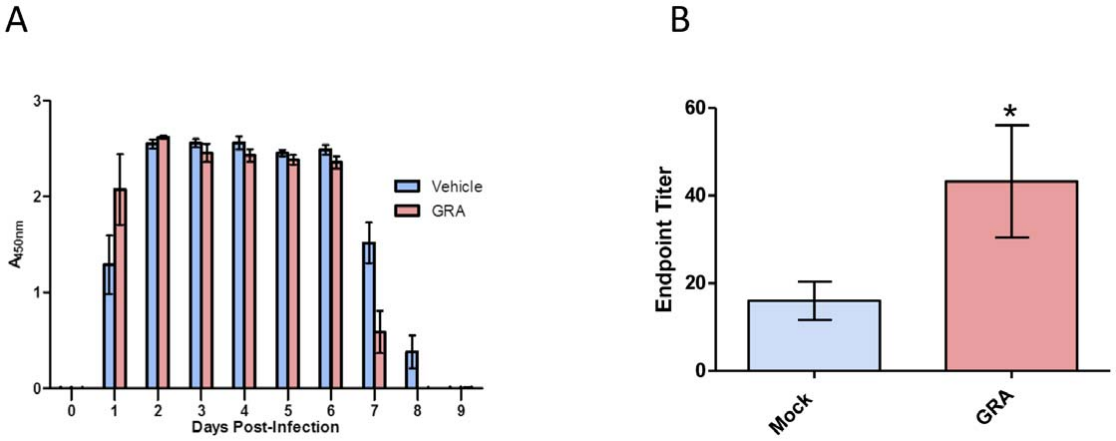
GRA reduces the duration of rotavirus antigen shedding. C57Bl/6 mice (n = 5 mice per group) were administered 50 mg/kg GRA or vehicle alone by oral gavage one day pre-infection and then one day post-infection. A) Fecal samples from individual mice were collected and rotavirus antigen was detected by ELISA. B) Total endpoint anti-rotavirus serum antibody titers were measured by ELISA. Error bars are SEM; p = 0.03.

We reported that GRA inhibits rotavirus replication in cell culture [23], [30]. The ability of GRA to attenuate virus replication *in vivo* was tested. In the adult mouse model of rotavirus infection, the magnitude of replication is measured by fecal antigen shedding [20], [31]. Mice were administered GRA or vehicle by oral gavage one day pre-infection with EW, and then again one day post-infection. The day of onset and magnitude of virus shedding was not different between GRA-treated and vehicle-treated animals (Figure 6A). However, virus shedding in mice that received GRA was resolved one day earlier, and on the last positive day was reduced by approximately 50%. Viral antigen was not detectable in either group at day nine post-infection. GRA treatment did not result in lower amounts of fecal antigen, but shortened the duration of virus shedding, suggesting an effect of GRA on the immune response to infection instead of a direct effect on virus replication. This idea is supported by the observation that anti-rotavirus serum antibody titers were statistically higher in GRA-treated animals relative to controls, although the difference was small (Figure 6B). Anti-rotavirus fecal IgA titers were not different between treated and untreated animals (data not shown).

### GRA Enhances Accumulation of CD3^+^ T Cells in the PPs of Rotavirus-infected Mice Early Post-infection

**Figure 7:**
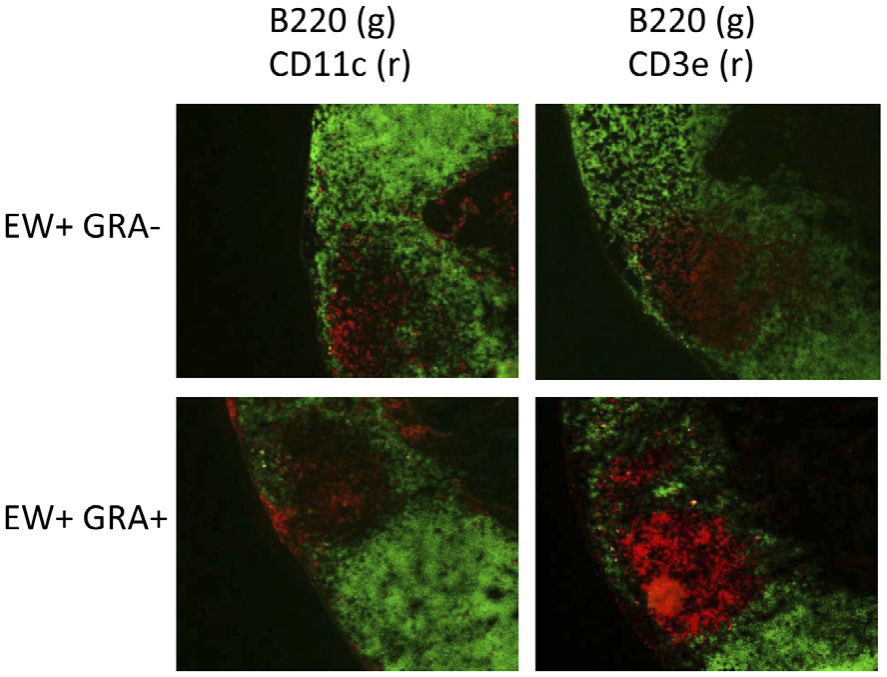
GRA induces T cell expansion in PPs of rotavirus-infected mice. PP tissue sections from EW-infected mice from the experiment described in the legend to Figure 4 were stained for the detection of B cells (B220), DCs (CD11c), and T cells (CD3). Magnification = 10x.

PPs from infected mice treated with GRA or vehicle from the same experiment described above harvested at the early time point were evaluated for changes in B220, CD11c, and CD3 expression. There were marked increases in CD3^+^ T cells in the PPs of infected mice administered GRA compared to vehicle-treated or uninfected mice (Figure 7), suggesting GRA enhances T cell accumulation in mucosal inductive sites in response to infection, a correlation supported by flow cytometry (Figures 1 and 3).

## Discussion

Most *in vivo* studies that describe immune system modulating activity of licorice root-derived compounds GA and GRA have used intraperitoneal, subcutaneous, or intravenous routes of administration either in human patients or in animal models. Exceptions include studies of the effects of GA, GRA or other bioactive components of licorice in mouse models of allergy, or oral and gastric ulcerative lesions [1], [13], [14]. There are no studies of which we are aware that analyze extended immunomodulatory gene expression and cellular responses induced at the gut mucosa upon oral delivery of GRA.

The pattern of chemokine and chemokine receptor transcript expression induced by GRA is consistent with that described for immune cell recruitment to the gut and maturation of ILFs [24], [25]. Analysis of cell populations associated with ILF formation in transgenic mice engineered to express CXCL13 in intestinal epithelial cells indicated a mechanism of CXCL13-mediated recruitment of B cells, as well as lymphoid tissue inducer (LTi)-like and NK cells to the gut mucosa [24]. In addition to the role of CXCL13 in ILF expansion and relevant to the gene expression induced by GRA, TLR activated LTi-like express LTα1LTβ2 to interact with the LTβR on stromal cells, which in turn release cytokines including DC recruitment ligands CCL19 and CCL21, that together with other signals including IL-22, result in maturation of ILF and a T cell independent B cell response [32], [33]. GRA-induced gene expression data presented here thus are consistent with the roles of CXCL13, CXCR5, CCR6, CCR7, CCL19, CCL21, and LTA/LTB in ILF formation [24], [25], [32], [34]. That the pattern of GRA-mediated transcript induction is functionally relevant is further supported by increases in CD19^+^ cells in the LP, and presence of mature ILF in GRA-treated mice. The same pattern of gene expression induced by GRA was observed when mice were given the parent compound GA (data not shown). Although rapidly metabolized by gut commensal bacteria, GA is present in the natural licorice extract in the highest concentration. It will be important to investigate whether genes up-regulated by purified GRA and cell recruitment to the gut also are modulated by crude extract commonly used a dietary supplement.

ILFs arise from precursor cryptopatches upon luminal stimuli, and their development and maturation are dependent on both dietary ligands and post-gestational acquisition of gut microbiota [32], [35]–[38]. A critical and required role for the aryl hydrocarbon receptor (AhR) in regulating ILF maturation recently has been reported [38]. AhR is a ligand-activated transcription factor responsive to environmental signals including xenobiotics, dietary and endogenous ligands [39]. AhR activation results in signaling and gene expression patterns that regulate multiple physiological processes including detoxification, immune cell modulation and maintenance of metabolic homeostasis. AhR^−/−^ mice or mice fed a diet deficient in AhR ligands do not develop ILF, and ILF are restored by addition of an AhR ligand to deficient diets [38]. Studies to determine whether GRA or other components of licorice extract act through the AhR and thus introduce a new ligand for this receptor are ongoing.

ILF are dynamic structures particularly responsive to changes in gut flora, and play a central role in regulating IgA production that controls commensal populations [40]. The dependence of ILF formation on the composition of the microbiota puts forth the intriguing possibility that GRA alters the composition of the bacterial population in the gut. Recognition of bacterial peptidoglycan by pattern recognition receptor NOD1 in epithelial cells also is required for optimal ILF formation, [35], putting forth an alternative hypothesis that GRA activates signaling pathways controlled by NOD1 and TLR, thus offering an explanation for the rapid gene induction. Whether GRA, GA or crude licorice root extracts affect the interplay between gut tissue and the microbiota that could be responsible for some of the immune system modulating effects that have been attributed to these compounds warrants investigation.

Oral administration of GRA to mice one day prior to and one day after infection with rotavirus did not affect the onset or magnitude of fecal antigen shedding, but shedding resolved more than one day sooner compared to untreated animals. The lack of a difference between onset and magnitude of virus replication supports the idea that effects of GRA in the infected mouse are immune-mediated, as administration of GRA was associated with accelerated clearance. Whether the reduction in the duration of shedding is a direct result of ILF maturation is under investigation. Notably, GRA induced CD19^+^ cell accumulation in the LP, and ILF formation in the LP of both uninfected and infected mice, suggesting GRA affects signaling pathways that drive lymphocyte recruitment, and can occur independently of virus infection. ILF regulate IgA production to maintain intestinal homeostasis as well as to respond effectively to pathogens. A defined role for these ILF in rotavirus clearance remains to be determined. GRA also had an effect on expansion of T cells in the PP early post-infection, suggesting GRA is pleotropic in its ability to modulate immune cell activity. Detailed mechanisms by which GRA induces these responses at the gut mucosa, including identification of target cells currently are under investigation.

## References

[1] Asl MN, Hosseinzadeh H (2008) Review of pharmacological effects of Glycyrrhiza sp. and its bioactive compounds. Phytother Res 22: 709–724. 18446848 10.1002/ptr.2362 PMC7167813

[2] Schrofelbauer B, Raffetseder J, Hauner M, Wolkerstorfer A, Ernst W, et al (2009) Glycyrrhizin, the main active compound in liquorice, attenuates pro-inflammatory responses by interfering with membrane-dependent receptor signalling. Biochem J 421: 473–482. 19442240 10.1042/BJ20082416

[3] Wolkerstorfer A, Kurz H, Bachhofner N, Szolar OH (2009) Glycyrrhizin inhibits influenza A virus uptake into the cell. Antiviral Res 83: 171–178. 19416738 10.1016/j.antiviral.2009.04.012 PMC7126985

[4] Menegazzi M, Di Paola R, Mazzon E, Genovese T, Crisafulli C, et al (2008) Glycyrrhizin attenuates the development of carrageenan-induced lung injury in mice. Pharmacol Res 58: 22–31. 18590825 10.1016/j.phrs.2008.05.012

[5] Armanini D, Fiore C, Mattarello MJ, Bielenberg J, Palermo M (2002) History of the endocrine effects of licorice. Experimental and Clinical Endocrinology and Diabetes 110: 257–261. 12373628 10.1055/s-2002-34587

[6] Fiore C, Eisenhut M, Krausse R, Ragazzi E, Pellati D, et al (2008) Antiviral effects of Glycyrrhiza species. Phytother Res 22: 141–148. 17886224 10.1002/ptr.2295 PMC7167979

[7] van Rossum TG, Vulto AG, de Man RA, Brouwer JT, Schalm SW (1998) Review article: glycyrrhizin as a potential treatment for chronic hepatitis C. Aliment Pharmacol Ther. 12: 199–205. 10.1046/j.1365-2036.1998.00309.x 9570253

[8] Orlent H, Hansen BE, Willems M, Brouwer JT, Huber R, et al (2006) Biochemical and histological effects of 26 weeks of glycyrrhizin treatment in chronic hepatitis C: a randomized phase II trial. J Hepatol 45: 539–546. 16905220 10.1016/j.jhep.2006.05.015

[9] Ikeda K, Arase Y, Kobayashi M, Saitoh S, Someya T, et al (2006) A long-term glycyrrhizin injection therapy reduces hepatocellular carcinogenesis rate in patients with interferon-resistant active chronic hepatitis C: a cohort study of 1249 patients. Dig Dis Sci 51: 603–609. 16614974 10.1007/s10620-006-3177-0

[10] Arase Y, Ikeda K, Murashima N, Chayama K, Tsubota A, et al (1997) The long term efficacy of glycyrrhizin in chronic hepatitis C patients. Cancer 79: 1494–1500. 9118029 10.1002/(sici)1097-0142(19970415)79:8<1494::aid-cncr8>3.0.co;2-b

[11] Utsunomiya T, Kobayashi M, Pollard RB, Suzuki F (1997) Glycyrrhizin, an active component of licorice roots, reduces morbidity and mortality of mice infected with lethal doses of influenza virus. Antimicrob Agents Chemother 41: 551–556. 9055991 10.1128/aac.41.3.551 PMC163749

[12] Ni YF, Kuai JK, Lu ZF, Yang GD, Fu HY, et al (2011) Glycyrrhizin treatment is associated with attenuation of lipopolysaccharide-induced acute lung injury by inhibiting cyclooxygenase-2 and inducible nitric oxide synthase expression. J Surg Res 165: e29–35. 21074783 10.1016/j.jss.2010.10.004

[13] Shin YW, Bae EA, Lee B, Lee SH, Kim JA, et al (2007) In vitro and in vivo antiallergic effects of Glycyrrhiza glabra and its components. Planta Med 73: 257–261. 17327992 10.1055/s-2007-967126

[14] Sun Y, Cai TT, Shen Y, Zhou XB, Chen T, et al (2009) Si-Ni-San, a traditional Chinese prescription, and its active ingredient glycyrrhizin ameliorate experimental colitis through regulating cytokine balance. Int Immunopharmacol 9: 1437–1443. 19733696 10.1016/j.intimp.2009.08.017

[15] Di Paola R, Menegazzi M, Mazzon E, Genovese T, Crisafulli C, et al (2009) Protective effects of glycyrrhizin in a gut hypoxia (ischemia)-reoxygenation (reperfusion) model. Intensive Care Med 35: 687–697. 18953525 10.1007/s00134-008-1334-y

[16] Hattori M, Sakamoto T, Kobashi K, Namba T (1983) Metabolism of glycyrrhizin by human intestinal flora. Planta Med 48: 38–42. 6611743 10.1055/s-2007-969875

[17] Ukil A, Biswas A, Das T, Das PK (2005) 18 Beta-glycyrrhetinic acid triggers curative Th1 response and nitric oxide up-regulation in experimental visceral leishmaniasis associated with the activation of NF-kappa B. J Immunol. 175: 1161–1169. 10.4049/jimmunol.175.2.1161 16002718

[18] Li HE, Qiu JZ, Yang ZQ, Dong J, Wang JF, et al (2012) Glycyrrhetinic acid protects mice from Staphylococcus aureus pneumonia. Fitoterapia 83: 241–248. 22085765 10.1016/j.fitote.2011.10.018

[19] Long DRM J, Hendricks JM, Hardy ME, Voyich JM (2012) 18beta-glycyrrhetinic acid inhibits MRSA survival and attenuates virulence gene expression. Antimicrob Agents Chemother accepted pending revision. 10.1128/AAC.01023-12 PMC3535912 23114775

[20] Burns JW, Krishnaney AA, Vo PT, Rouse RV, Anderson LJ, et al (1995) Analyses of homologous rotavirus infection in the mouse model. Virology 207: 143–153. 7871723 10.1006/viro.1995.1060

[21] Csencsits KL, Walters N, Pascual DW (2001) Cutting edge: dichotomy of homing receptor dependence by mucosal effector B cells: alpha(E) versus L-selectin. J Immunol 167: 2441–2445. 11509580 10.4049/jimmunol.167.5.2441

[22] O’Neal CM, Crawford SE, Estes MK, Conner ME (1997) Rotavirus virus-like particles administered mucosally induce protective immunity. J Virol 71: 8707–8717. 9343229 10.1128/jvi.71.11.8707-8717.1997 PMC192335

[23] Shaneyfelt ME, Burke AD, Graff JW, Jutila MA, Hardy ME (2006) Natural products that reduce rotavirus infectivity identified by a cell-based moderate-throughput screening assay. Virol J 3: 68. 16948846 10.1186/1743-422X-3-68 PMC1564392

[24] Marchesi F, Martin AP, Thirunarayanan N, Devany E, Mayer L, et al (2009) CXCL13 expression in the gut promotes accumulation of IL-22-producing lymphoid tissue-inducer cells, and formation of isolated lymphoid follicles. Mucosal Immunol 2: 486–494. 19741597 10.1038/mi.2009.113

[25] Velaga S, Herbrand H, Friedrichsen M, Jiong T, Dorsch M, et al (2009) Chemokine receptor CXCR5 supports solitary intestinal lymphoid tissue formation, B cell homing, and induction of intestinal IgA responses. J Immunol 182: 2610–2619. 19234155 10.4049/jimmunol.0801141

[26] Sanderson RD, Lalor P, Bernfield M (1989) B lymphocytes express and lose syndecan at specific stages of differentiation. Cell Regul 1: 27–35. 2519615 10.1091/mbc.1.1.27 PMC361422

[27] Hamada H, Hiroi T, Nishiyama Y, Takahashi H, Masunaga Y, et al (2002) Identification of multiple isolated lymphoid follicles on the antimesenteric wall of the mouse small intestine. J Immunol 168: 57–64. 11751946 10.4049/jimmunol.168.1.57

[28] Eberl G (2005) Inducible lymphoid tissues in the adult gut: recapitulation of a fetal developmental pathway? Nat Rev Immunol 5: 413–420. 15841100 10.1038/nri1600

[29] Pabst O, Herbrand H, Worbs T, Friedrichsen M, Yan S, et al (2005) Cryptopatches and isolated lymphoid follicles: dynamic lymphoid tissues dispensable for the generation of intraepithelial lymphocytes. Eur J Immunol 35: 98–107. 15580658 10.1002/eji.200425432

[30] Hardy ME, Hendricks JM, Paulson JM, Faunce NR (2012) 18beta-glycyrrhetinic acid inhibits rotavirus replication in culture. Virol J 9: 96. 22616823 10.1186/1743-422X-9-96 PMC3478227

[31] Rose J, Franco M, Greenberg H (1998) The immunology of rotavirus infection in the mouse. Adv Virus Res 51: 203–235. 9891588 10.1016/s0065-3527(08)60786-1

[32] Lorenz RG, Chaplin DD, McDonald KG, McDonough JS, Newberry RD (2003) Isolated lymphoid follicle formation is inducible and dependent upon lymphotoxin-sufficient B lymphocytes, lymphotoxin beta receptor, and TNF receptor I function. J Immunol 170: 5475–5482. 12759424 10.4049/jimmunol.170.11.5475

[33] Cerutti A, Puga I, Cols M (2011) Innate control of B cell responses. Trends Immunol 32: 202–211. 21419699 10.1016/j.it.2011.02.004 PMC3090458

[34] McDonald KG, McDonough JS, Newberry RD (2005) Adaptive immune responses are dispensable for isolated lymphoid follicle formation: antigen-naive, lymphotoxin-sufficient B lymphocytes drive the formation of mature isolated lymphoid follicles. J Immunol 174: 5720–5728. 15843574 10.4049/jimmunol.174.9.5720

[35] Bouskra D, Brezillon C, Berard M, Werts C, Varona R, et al (2008) Lymphoid tissue genesis induced by commensals through NOD1 regulates intestinal homeostasis. Nature 456: 507–510. 18987631 10.1038/nature07450

[36] Suzuki K, Fagarasan S (2008) How host-bacterial interactions lead to IgA synthesis in the gut. Trends Immunol 29: 523–531. 18838301 10.1016/j.it.2008.08.001

[37] Fagarasan S, Muramatsu M, Suzuki K, Nagaoka H, Hiai H, et al (2002) Critical roles of activation-induced cytidine deaminase in the homeostasis of gut flora. Science 298: 1424–1427. 12434060 10.1126/science.1077336

[38] Kiss EA, Vonarbourg C, Kopfmann S, Hobeika E, Finke D, et al (2011) Natural aryl hydrocarbon receptor ligands control organogenesis of intestinal lymphoid follicles. Science 334: 1561–1565. 22033518 10.1126/science.1214914

[39] Barouki R, Aggerbeck M, Aggerbeck L, Coumoul X (2012) The aryl hydrocarbon receptor system. Drug Metabol Drug Interact 27: 3–8. 22718620 10.1515/dmdi-2011-0035

[40] Knoop KA, Newberry RD (2012) Isolated Lymphoid Follicles are Dynamic Reservoirs for the Induction of Intestinal IgA. Front Immunol 3: 84. 22566964 10.3389/fimmu.2012.00084 PMC3343265

